# Influence of various reproduction substrates on the survival, health, and reproductive output of broodstock Mediterranean medicinal leech (*Hirudo verbana*)

**DOI:** 10.3389/fvets.2026.1969385

**Published:** 2026-09-24

**Authors:** Mustafa Ceylan

**Affiliations:** 1 Faculty of Egirdir Fisheries, Isparta University of Applied Sciences, Isparta, Türkiye; 2 Medicinal Leech Application and Research Center, Isparta University of Applied Sciences, Isparta, Türkiye

**Keywords:** conservation breeding, electrical conductivity, Hirudinea, horticultural growing media, non-conventional species aquaculture, osmotic stress

## Abstract

The captive breeding of non-traditional aquatic species is becoming increasingly important for both niche animal production and the conservation of overexploited taxa; however, breeding protocols for these species remain largely undocumented. The Mediterranean medicinal leech (*Hirudo verbana*), which is clinically used in reconstructive surgery and listed on CITES Appendix II, deposits its cocoons on a moist terrestrial substrate; this makes the reproductive substrate a critical but untested management variable. We compared four substrates available to leech breeders, namely unamended fresh lake peat (FLP), unamended commercial terrestrial peat (CTP), commercial lake peat (CLP), and vermicompost (VC). Each substrate was characterized across 14 physicochemical parameters and evaluated for its effects on the survival, health, and reproductive output of broodstock leeches. Substrate type significantly altered survival (log-rank χ^2^(3) = 53.3, *P* < 0.001): all leeches died within 2 days in VC and within 4 days in CLP; in contrast, 50% (FLP) and 60% (CTP) survived the entire 75-day experiment. Principal component analysis and hierarchical clustering distinguished lethal substrates from viable substrates along an ionic and osmotic gradient driven by electrical conductivity up to 10.14 dS/m and high levels of sodium, copper, and zinc, indicating that the immediate cause of death was ionic and osmotic stress rather than organic richness. No cocoons were produced on the lethal substrates, whereas on the two viable substrates, the number of cocoons, cocoon morphology, number of offspring per cocoon, and offspring body weight were equivalent. These findings reveal that commercially processed horticultural growing media pose an uncharacterized acute threat to captive reproduction and support the use of electrical conductivity as a low-cost, quantitative pre-use screening criterion; this principle is likely applicable to other exotic and wild species reared in semi-terrestrial breeding environments as well.

## 1 Introduction

In veterinary medicine, reproductive science relies heavily on traditional domestic vertebrates and generally leaves reproductive protocols for aquatic, exotic, and wild species to artisanal practice rather than rigorous experimental evidence (1, 2). The Mediterranean medicinal leech is an instructive case in point, because it sits directly at the interface of animal and human health. These annelid worms have returned to mainstream clinical practice as an established adjunct in reconstructive and microvascular surgery, and their biologically active salivary secretions continue to attract pharmaceutical interest (3, 4). Their applications now extend to veterinary medicine as well, where they provide a non-invasive blood collection method for mammals under human care (5). These applications in the clinical, pharmaceutical, and veterinary fields are reviving demand for these animals. That demand has fallen largely on wild populations, and sustained over-collection together with wetland loss has driven the principal European taxa, *Hirudo medicinalis* Linnaeus, 1758 and the Mediterranean *Hirudo verbana* Carena, 1820, into a conservation-dependent status under CITES Appendix II and national legislation (6, 7).

Recent molecular and biogeographic surveys across the European and Mediterranean range confirm that these species now persist as fragmented, genetically structured populations of conservation concern (8–10), and quantitative assessments of individual wild stocks report densities too low to sustain continued commercial collecting (11). Therefore, we argue that controlled captive reproduction is no longer a purely commercial ambition, but a fundamental prerequisite for relieving harvesting pressure on natural leech stocks while still supplying clinical and industrial users with leeches of known provenance and health status.

Despite this urgency, leech aquaculture remains an incompletely codified husbandry system, and several of its critical steps continue to rely on artisanal practice rather than on experimental evidence (12, 13). The reproductive biology of *Hirudo* is unusual among cultured aquatic animals and explains why the rearing environment is so influential. Although mating occurs within the aquatic environment, these hermaphroditic annelids migrate to damp terrestrial or semi-terrestrial substrates to deposit their cocoons. The deposited cocoons remain in this moist substrate throughout their incubation and embryogenesis, culminating in the emergence of offspring that subsequently re-enter the water (14), and the European *Hirudo* species differ markedly in fecundity and reproductive strategy (15). Consequently, the substrate provided during this phase is not merely an inert medium, but rather a dynamic physical and chemical matrix surrounding the gravid adult and its developing cocoon. A growing body of work has shown that reproductive success in *H. verbana* responds sensitively to husbandry variables such as broodstock density (16, 17), maternal age and body size (18), blood meal source, frequency, and composition (13, 19, 20), photoperiod (21), and cocoon incubation angle (22). Furthermore, research into alternative models for leech production shows promise (23). By comparison, the reproduction substrate itself has received little systematic attention, even though it is the variable that producers manipulate most freely and least consistently.

In commercial practice, the substrates used for leech reproduction are drawn almost entirely from the horticultural supply chain, where peats, composts, and vermicompost are selected and processed to maximize plant growth. To that end, they are frequently enriched with mineral fertilizers and organic amendments that raise nutrient concentrations, salinity, and micronutrient metal content far above the levels found in the natural wetland sediments that leeches encounter in the wild (24). There is a strong biological basis to expect that such enrichment could be harmful. *H. verbana* is a freshwater osmoregulator with documented sensitivity to dissolved ions and metals, particularly to copper and zinc, which have been demonstrated in controlled toxicological studies to be lethal to this species at environmentally realistic concentrations (25, 26). More broadly, salinity, and the electrical conductivity that indexes it, are established determinants of survival and community composition in freshwater invertebrates (27, 28). The porous media used for reproduction also interact directly with leech attachment and locomotion (29), so substrate properties may influence the animals through several simultaneous pathways. Field observations reinforce the point, because leeches in nature select specific oviposition microhabitats such as damp, organically rich lake margins and even the nests of waterfowl, which suggests that the physicochemical envelope of the reproduction substrate is ecologically constrained (30). Leech breeders themselves have also reported sudden mass die-offs and cases of cocoon failure in certain substrate batches; this situation is consistent with an undetected chemical hazard. However, to our knowledge, the phenomenon has not been characterized experimentally, and the substrate properties responsible have not been identified. Since such losses stem from a single routine decision made in husbandry, they raise not only an economic issue but also a welfare issue.

The few studies that have compared rearing environments for medicinal leeches have generally treated the reproductive substrate as a categorical husbandry choice and have not linked biological outcomes to measured substrate chemistry (31–33). As a result, producers lack both a mechanistic understanding of why some substrates fail and a simple, measurable criterion by which a substrate can be screened before valuable broodstock is committed to it. Our laboratory's own experience illustrates the problem: the substrate used for our broodstock was settled on the basis of accumulated practical experience rather than measured evidence, after losses on media that had never been characterized chemically before use. Closing the gap between husbandry practice and measured substrate chemistry requires an approach that couples a survival and reproduction bioassay with a multivariate description of substrate physicochemistry, so that the biological response can be mapped onto the chemical gradients that distinguish candidate substrates.

The present study was designed to provide that coupling. To the best of our knowledge, this work represents the first systematic comparison of four reproduction substrates that represent the primary options available to producers, namely unamended fresh lake peat collected from a natural leech habitat, an unamended commercial terrestrial peat, a commercial lake peat, and a horticultural vermicompost, evaluating their effects on the survival, reproductive output, cocoon morphometry, and health of broodstock Mediterranean medicinal leech, *H. verbana*. We characterized each substrate for 14 physicochemical parameters to identify the key properties associated with reproductive suitability. Because two substrates caused rapid and complete mortality before any cocoons were produced, the reproductive comparisons necessarily focused on the two viable substrates. We hypothesized that survival and reproductive performance would depend largely on substrate identity. Specifically, we proposed that the primary distinguishing factor for substrate suitability is ionic and osmotic loading rather than organic richness. Consequently, we investigated whether routinely measurable properties, such as electrical conductivity, could serve as practical screening tools for leech farming and, more generally, as transferable criteria for species reared in semi-terrestrial breeding environments.

## 2 Materials and methods

### 2.1 Study site and leeches

The present 75-day study was conducted at the laboratory of the Medicinal Leech Application and Research Center, Isparta University of Applied Sciences, Türkiye. Adult, sexually mature *H. verbana* were obtained from the center's controlled laboratory broodstock. Before the trial the animals were acclimated for 10 days in 5-L jars containing 4 L of dechlorinated water at a stocking density of approximately 8 g leech biomass per liter and at 23–24 °C, under the natural seasonal light regime prevailing during the reproductive season rather than an imposed artificial photoperiod; the water was renewed every other day. In accordance with the routine practice of this laboratory and of commercial leech producers, every broodstock individual received its final blood meal 45 days before being transferred to the reproduction substrates, and no animal was fed at any point during the 75-day trial. This pre-transfer interval is required because leeches moved from the aquatic phase to a terrestrial reproduction substrate before digestion has sufficiently progressed regurgitate their blood meal and die. Only gravid individuals in good condition were enrolled. Each leech was weighed individually with a precision of 0.01 g at the start of the experiment to provide an initial body weight covariate, because body weight of broodstock leeches is a known determinant of reproductive output in medicinal leeches (34). The mean initial body weight was 7.64 ± 0.34 g (range: 4.92–16.64 g).

### 2.2 Substrates and experimental design

Four reproduction substrates were evaluated: unamended fresh lake peat (FLP), unamended commercial terrestrial peat (CTP), commercial lake peat (CLP), and vermicompost (VC). The FLP was obtained fresh from a licensed producer operating at Lake Çapali (Afyonkarahisar, Türkiye), a natural habitat of the medicinal leech *H. verbana*. CTP was procured from a licensed terrestrial peat extraction facility in Antalya (Türkiye). Finally, CLP and VC were sourced locally in Isparta (Türkiye) from a commercial horticultural supplier and a California red worm (*Eisenia fetida*) vermiculture facility, respectively.

A total of 40 gravid leeches were randomly assigned to four different substrate treatments (*n* = 10 per group). Prior to the experiment, a group comparison was conducted to ensure there was no significant difference in initial body weight among the groups (Kruskal-Wallis test: *P* = 0.784). Leeches were maintained individually in 1-L containers filled to approximately 75% capacity with the respective moistened substrate and kept at a controlled temperature of 23.3 ± 1.3 °C during the study. The containers were monitored daily for incidences of blood regurgitation and mortality, and weekly for cocoon deposition. During the weekly cocoon checks, substrate moisture was assessed and, where necessary, restored with a hand sprayer so as to hold all containers at approximately 70%−75% moisture throughout the trial, following Ceylan et al. (16). Any occurrences of mortality or visible morphological deformities were systematically recorded along with their exact dates of observation. Furthermore, the total number of cocoons produced per individual and the terminal body weight were meticulously documented.

CTP is the medium that our laboratory has used for its broodstock leeches for several years, a choice settled empirically rather than on measured criteria, and it therefore entered the trial as the de facto standard of current practice. Fresh lake peat, collected from a natural *H. verbana* habitat, provided the corresponding natural-habitat reference. Both were run as full experimental groups of 10 individually housed animals, so the comparison is internally anchored to current practice and to the natural condition without requiring a separate external control.

### 2.3 Substrate physicochemical analysis

Each of the four substrates was characterized by 14 physicochemical parameters spanning macronutrients, micronutrient metals, and bulk physical properties. These parameters encompassed total nitrogen, organic matter, and moisture; a suite of macro and trace elements including sodium, calcium, magnesium, potassium, phosphorus, zinc, iron, copper, and manganese; alongside electrical conductivity and pH. A single composite sample of each substrate, drawn from the same batch that was used to fill the experimental containers, was submitted for analysis. All analyses, including electrical conductivity and pH, were performed on oven-dried material, so every value in Table 1 is expressed on a dry-matter basis and the four substrates are directly comparable; conductivity and pH were determined on a 1:5 (w/v) substrate-to-deionized-water extract of that oven-dried material, and conductivity values should therefore be compared only with measurements made on the same basis. The moisture percentages in Table 1 are an independent descriptive property of the source materials as received and do not enter the determination of any other parameter; substrate moisture during the trial itself was held at 70%−75% in every container, as described in Section 2.2. All physicochemical analyses were conducted at the Isparta Soil and Plant Analysis Laboratory (ISLAB, Isparta, Türkiye), a commercial facility officially accredited for basic soil analysis by the Republic of Türkiye Ministry of Agriculture and Forestry. These specific parameters were selected because they effectively capture the nutrient enrichment, salinity, and metal loading by which horticulturally processed media are expected to differ from natural wetland sediments (24). Furthermore, copper and zinc were included specifically due to their documented toxicity to *H. verbana* (25).

**Table 1:** Physicochemical characterization of the four reproductive substrates used in the study.

Parameter	Unit	Commercial terrestrial peat (CTP)	Fresh lake peat (FLP)	Commercial lake peat (CLP)	Vermicompost (VC)
Total nitrogen	%	2.5	0.8	1.6	1.4
Phosphorus	ppm	633	530	1,927	6,223
Potassium	ppm	1,549	2,354	844	13,670
Calcium	ppm	53,617	80,060	48,056	47,279
Magnesium	ppm	8,073	20,170	20,509	10,584
Sodium	ppm	749	361	1,072	6,166
Iron	Ppm	8,157	9,637	7,909	4,701
Copper	ppm	13.52	9.41	37.87	42.7
Manganese	ppm	124.96	165.09	447.49	397.44
Zinc	ppm	56.30	38.27	135.6	142.55
Organic matter	%	66.26	34.74	45.74	39.68
Moisture	%	66.72	51.56	43.69	50.73
pH		7.24	7.50	7.39	8.53
Electrical conductivity	dS/m	3.69	1.19	9.47	10.14

All parameters were determined on oven-dried material and are therefore reported on a dry-matter basis; electrical conductivity and pH were measured on a 1:5 (w/v) substrate-to-deionised-water extract of the oven-dried sample. The moisture percentages are the as-received contents of the source materials and are reported as a descriptive property only. The acute lethal substrates (VC and CLP) are distinguished by profoundly elevated electrical conductivity and high concentrations of sodium, potassium, copper, and zinc compared to the viable substrates (CTP and FLP), indicating severe osmotic and ionic loading.

### 2.4 Cocoon morphometry and offspring measurements

Cocoons produced on the two substrates that supported reproduction were collected and measured individually. For each cocoon we recorded weight (g) and four external and internal dimensions, namely external and internal length and external and internal diameter (mm), and we counted the number of emerged offspring per cocoon. Each offspring was weighed individually with a precision of 0.1 mg. Of the 58 cocoons deposited in total, a subsample of 46 cocoons (FLP, *n* = 22; CTP, *n* = 24) was utilized for morphometric measurements. Offspring body weight was recorded for 366 offspring in total, comprising 218 offspring from 16 cocoons deposited by 7 mothers on FLP and 148 offspring from 13 cocoons deposited by 6 mothers on CTP.

### 2.5 Statistical analysis

All statistical computations and graphical representations were executed using the R programming environment. Results are expressed as the mean ± standard error (SE), with the threshold for statistical significance established at *P* < 0.05. Because two substrates caused complete pre-reproductive mortality, reproductive assessments were restricted to the two viable substrates (FLP and CTP). For each continuous reproductive and morphometric trait, normality and homogeneity of variance were assessed with the Shapiro–Wilk and Levene tests (stats and car packages, respectively), and an independent-samples Student *t*-test or a Mann–Whitney *U*-test was then selected on a per-trait basis according to the outcome of those checks; pregnancy duration, the number of offspring per cocoon, and the cocoon morphometric traits were compared in this way. Body weight loss over the trial, the onset of morphological deformation, and cocoon yield in animals that survived to day 75 compared with those that died earlier were analyzed in the same way, with the test chosen per trait according to the same normality and variance checks. The association between the onset of deformation and cocoon yield was assessed with Spearman's rank correlation. Cocoon yield per leech was analyzed using generalized linear models with a log link and Poisson or negative binomial error distributions, depending on overdispersion (MASS package); the Pearson dispersion statistic was 0.72, so the Poisson model was retained, and initial body weight of broodstock leeches was included as a covariate, alongside an unadjusted model and a leave-one-out sensitivity analysis to account for high-leverage individuals. To avoid pseudoreplication and account for the nested structure of the data, offspring body weight was analyzed using a linear mixed-effects model (LMM) via the lme4 package. Substrate type was set as a fixed effect, with random intercepts included for mother identity and for cocoon identity nested within mother.

Survival and categorical health outcomes across all four experimental groups were evaluated using Kaplan–Meier survival curves compared with pairwise log-rank tests with Holm adjustment (survival and survminer packages). Group differences in binary outcomes (mortality, regurgitation, and morphological deformities) were analyzed using Fisher's exact test throughout, in preference to Pearson's Chi-square, because several expected cell counts were below five, with Holm corrections for pairwise contrasts. To characterize the physicochemical signatures of the substrates and identify drivers of lethality, standardized parameters were subjected to Principal Component Analysis (stats and factoextra packages) and hierarchically clustered heatmaps using Ward. D2 linkage (pheatmap package). Graphical representations were generated programmatically using ggplot2, with multi-panel figures assembled using patchwork; data handling used dplyr.

### 2.6 Ethical approval

Under both the EU Directive 2010/63/EU and Turkish Regulation on Animal Experimentation Ethics Committees, ethical approval is required only for non-human vertebrates and live cephalopods. Therefore, formal approval was not required for *Hirudo verbana* (an invertebrate annelid). Nonetheless, standard leech aquaculture guidelines were strictly followed throughout the study to ensure animal welfare. No procedures resulting in death or other forms of harm were performed. The animals were monitored throughout a normal reproductive cycle that concluded at a predetermined experimental endpoint (the completion of the cycle in question). We specifically selected this period to mirror routine commercial breeding cycles within the medicinal leech industry, thereby establishing an experimental design with direct applicability to the sector. As severe pain or distress was not anticipated in this type of breeding comparison, a separate humane endpoint procedure was not predefined; we state this explicitly to avoid creating any contrary impression.

## 3 Results

### 3.1 Survival in leeches

Substrate identity had a large and highly significant effect on survival (log-rank test, χ^2^(3) = 53.3, *P* < 0.001; *n* = 10 leeches per substrate) (Figure 1). The two lake- and vermicompost-based commercial substrates were acutely lethal. All 10 leeches on CLP died within 4 days (mean death day, 2.7 ± 0.3), and all 10 on VC died within 2 days (mean death day, 1.4 ± 0.2). In contrast, the two-peat media that most closely resembled natural wetland sediment supported prolonged survival throughout the experimental period. 60 percent of leeches on CTP and 50% on FLP survived until the end of the 75-day experiment. Among the individuals that died on these substrates, mortality occurred much later in the experiment, with mean death days of 63.5 ± 6.0 for CTP and 50.2 ± 7.9 for FLP (Table 2).

**Figure 1:**
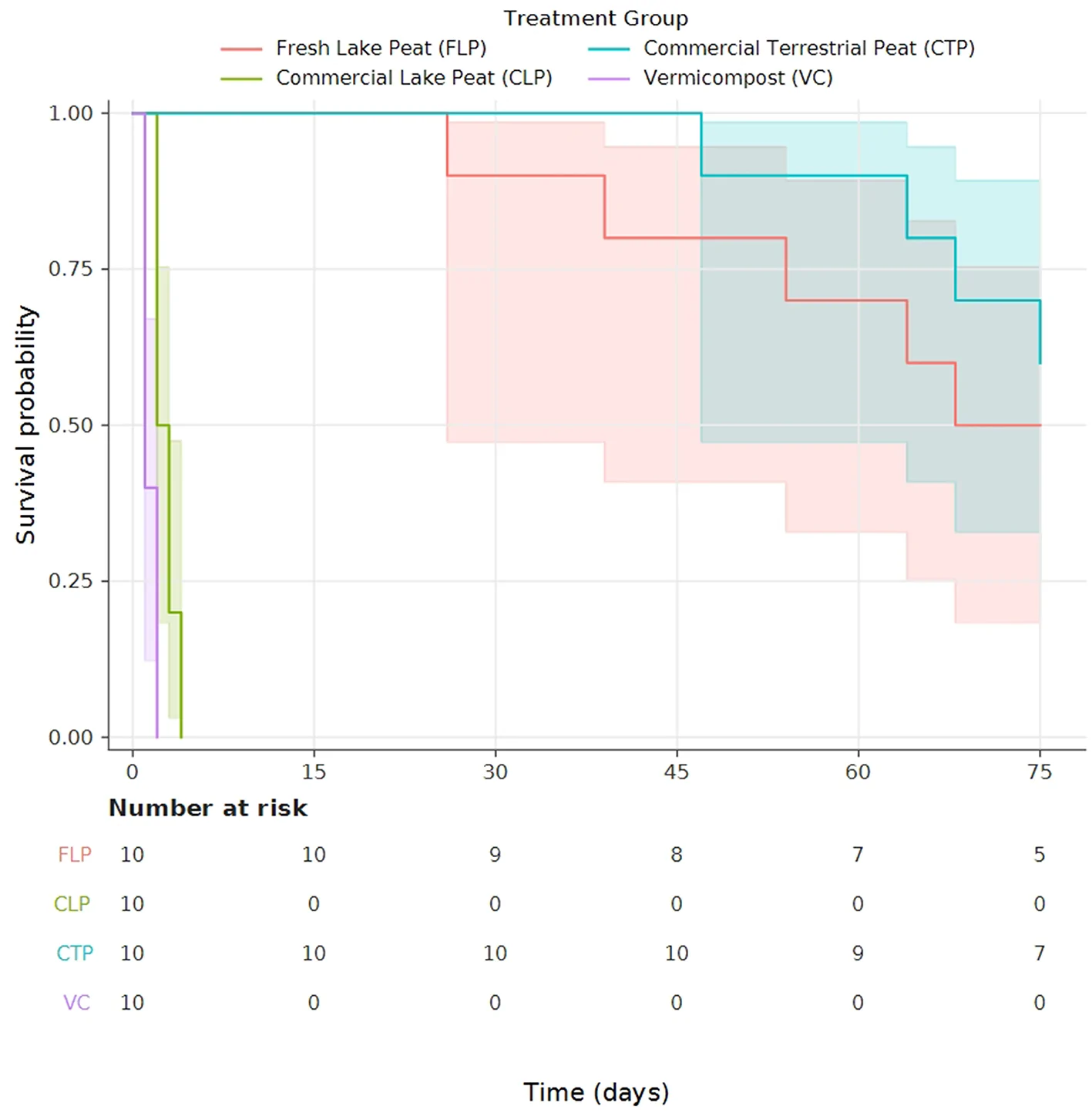
Kaplan–Meier survival curves of broodstock *Hirudo verbana* maintained on four reproductive substrates, with the corresponding number-at-risk table. Each leech was housed individually, so the experimental unit is the individual leech in its own container; *n* = 10 independent leeches (containers) per substrate at the start of the trial (40 in total). Survival differed significantly among substrate treatments (log-rank test: χ^2^(3) = 53.3, *P* < 0.001).

**Table 2:** Body weight, health parameters, and survival of *Hirudo verbana* broodstock maintained in different reproductive substrates.

Substrate	Initial body weight (g)	Final body weight (g)	Body weight loss (%)	Regurgitation incidence (%)	Incidence of deformation (%)	Onset of morphological deformation (days)	Onset of mortality (days)	Survival rate (%)
Commercial terrestrial peat (CTP)	7.72 ± 0.48	2.44 ± 0.25	67.3 ± 4.3	0	50	54.0 ± 7.7	63.5 ± 6.0	60
Fresh lake peat (FLP)	7.76 ± 1.02	3.11 ± 0.50	58.7 ± 5.3	10	70	27.4 ± 6.8	50.2 ± 7.9	50
Vermicompost (VC)	7.62 ± 0.59	–	–	40	–	–	1.4 ± 0.2	0
Commercial lake peat (CLP)	7.48 ± 0.63	–	–	30	–	–	2.7 ± 0.3	0
Sig.	0.784^a^	0.406^b^	0.082^b^	0.140^c^	0.650^c^	0.027^d^	< 0.001^a^	< 0.001^e^

Values are expressed as mean ± standard error. The experimental unit is the individual leech in its own 1-L container, and *n* = 10 per substrate for initial body weight, regurgitation incidence, onset of mortality, and survival rate. On the two viable substrates, final body weight was determined for all animals using their last available measurement prior to death or at the end of the trial (both CTP and FLP *n* = 10). In contrast, the onset of morphological deformation was recorded exclusively for affected individuals (CTP *n* = 5, FLP *n* = 7). Hyphens (-) indicate parameters that could not be evaluated due to the rapid mortality (100%) observed within the first few days in commercial lake peat (CLP) and vermicompost (VC). Superscript letters in the “Sig.” row indicate the statistical tests applied: ^a^Kruskal–Wallis test, ^b^Mann–Whitney U-test, ^c^Fisher's exact test, ^d^Student's t-test, ^e^Log-rank test.

The pairwise comparisons revealed a clear distinction between substrates that supported survival and those that did not. FLP and CTP each differed significantly from CLP and VC (Holm-adjusted *P* < 0.001), whereas the two viable substrates did not differ from one another (FLP vs. CTP, Holm-adjusted *P* = 0.512). The two lethal substrates also differed significantly, with survival being significantly shorter on VC than on CLP (Holm-adjusted *P* = 0.002). At the end of the 75-day experiment, the ultimate mortality proportions were consistent with the survival analysis (5/10 for FLP, 4/10 for CTP, and 10/10 for both CLP and VC; Fisher's exact test: *P* < 0.001).

### 3.2 Health status

Clinical signs of acute distress closely paralleled the observed mortality pattern. Blood regurgitation was most frequent in the lethal substrates, occurring in 4 of 10 leeches on VC and 3 of 10 on CLP, whereas it was rare or absent in the viable substrates (1 of 10 on FLP and none on CTP) (Table 2). Although this trend was biologically consistent with the survival outcomes, the difference was not statistically significant (Fisher's exact test, *P* = 0.140).

Morphological deformation could only be assessed in the two viable substrates because leeches maintained on VC and CLP died before deformities could develop. The incidence of deformation did not differ significantly between FLP (70%) and CTP (50%) (Fisher's exact test, *P* = 0.650). However, the onset of deformation occurred significantly earlier in FLP (27.4 ± 6.8 days) than in CTP (54.0 ± 7.7 days) (Student's *t*-test, *P* = 0.027), indicating that CTP not only supported survival but also delayed the development of morphological deformation (Table 2).

Among the twelve animals that developed morphological deformities, a later onset was associated with a higher cocoon yield (Spearman ρ = 0.804, *P* = 0.002). Because deformation onset could be timed only in animals that deformed, and because the number involved is small, this relationship is reported as exploratory; it is nonetheless consistent with the interpretation that morphological deformation marks a decline in broodstock condition that also curtails reproduction, and it indicates that reproductive output was not drawn disproportionately from animals already showing deformities.

Broodstock on both viable substrates lost a substantial proportion of their body weight over the 75-day non-feeding observation period. Mean body weight fell from 7.76 ± 1.02 g to 3.11 ± 0.50 g on FLP and from 7.72 ± 0.48 g to 2.44 ± 0.25 g on CTP, corresponding to an individual loss of 58.7 ± 5.3% (range 16.3%−76.6%) and 67.3 ± 4.3% (range 30.9%−76.7%) respectively. Neither the final body weight (Mann–Whitney *U*-test: *P* = 0.406) nor the proportional loss (Mann–Whitney *U*-test: *P* = 0.082) differed between the two viable substrates. The one leech that deposited no cocoons lost 16.3% of its body mass, against a mean of 65.5% in the nineteen animals that reproduced.

### 3.3 Reproduction performance

Because reproduction occurred only on FLP and CTP, the reproductive analyses compared these two substrates (*n* = 10 leeches per substrate). Cocoon production per leech was similar on the two substrates, at 2.7 ± 0.6 cocoons on FLP (27 cocoons in total) and 3.1 ± 0.4 on CTP (31 cocoons in total). The count of cocoons per leech was not overdispersed (dispersion 0.72), so a Poisson generalized linear model was retained. After adjustment for initial body weight of broodstock leeches, substrate had no detectable effect on cocoon number (incidence rate ratio for CTP relative to FLP 1.22, 95% confidence interval 0.72–2.06, *P* = 0.468). The same conclusion followed when the covariate was omitted (incidence rate ratio 1.15, 95% confidence interval 0.69–1.92, *P* = 0.600) and when the model was refitted without the most influential individual (incidence rate ratio 1.38, 95% confidence interval 0.76–2.52, *P* = 0.295). Initial body weight of broodstock leeches, by contrast, was a significant positive predictor, with each additional gram of initial body weight increasing the expected cocoon count by about 11% (incidence rate ratio 1.11, 95% confidence interval 1.02–1.21, *P* = 0.017). Similarly, the number of offspring did not differ significantly between the two substrates (Mann–Whitney *U*-test, *P* = 0.424), with 12.2 ± 1.2 offspring/cocoon produced on CTP compared to 13.5 ± 1.6 offspring/cocoon on FLP (Figure 2). Additionally, pregnancy duration was slightly longer on FLP (52.0 ± 2.9 days) than CTP (46.9 ± 2.7 days), but this difference was not statistically significant (Mann–Whitney *U*-test, *P* = 0.384). Offspring body weight behaved in the same way. Although the mean body weight of offspring produced on FLP (36.8 ± 1.0 mg) appeared slightly higher than those on CTP (33.7 ± 1.2 mg), this difference was not statistically significant when accounting for maternal and cocoon random effects (Linear mixed model: *P* = 0.587).

**Figure 2:**
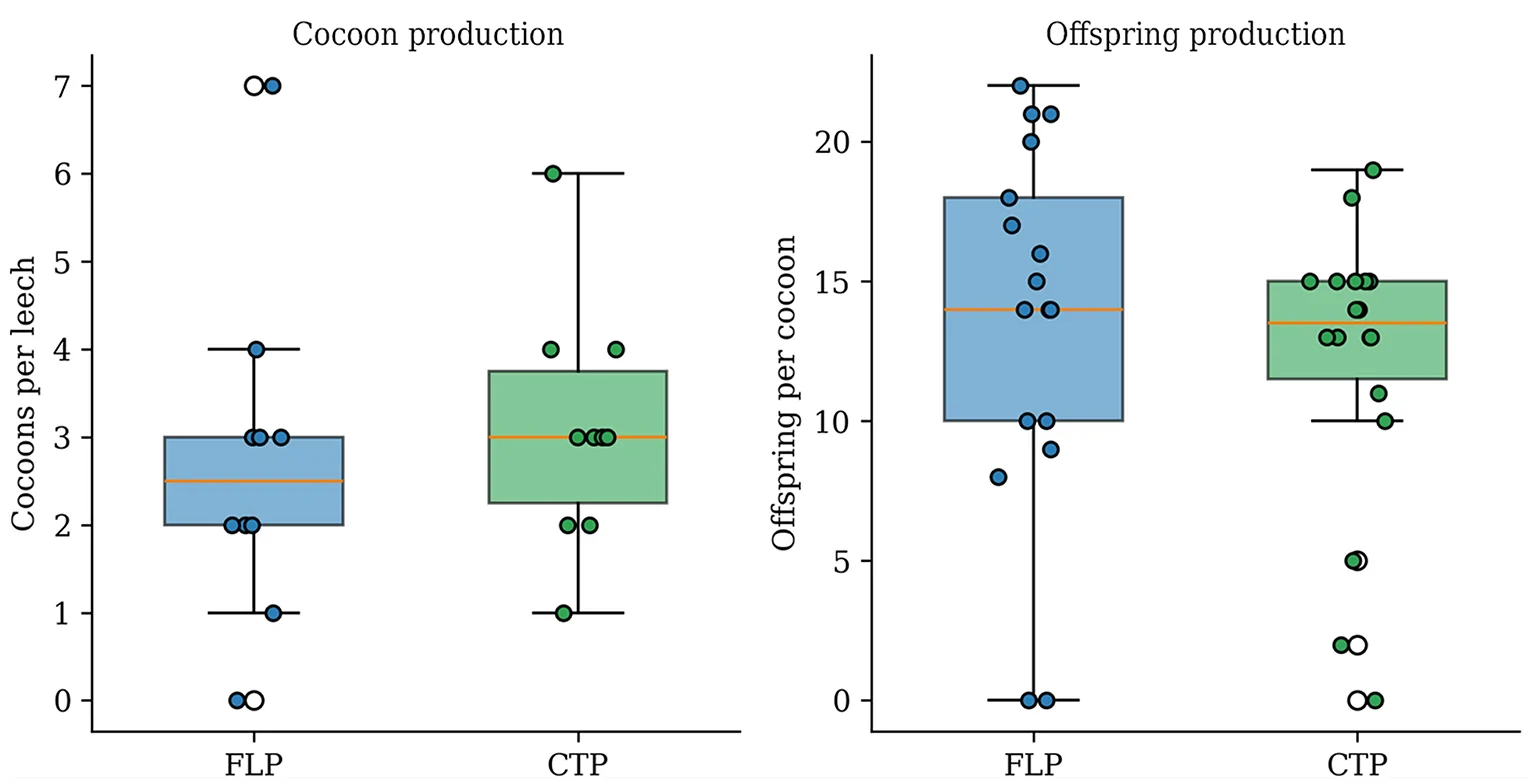
Cocoon production and offspring yield of *Hirudo verbana* on viable peat substrates, fresh lake peat (FLP) and commercial terrestrial peat (CTP). Cocoon production per leech was analyzed with the individual leech as the experimental unit (*n* = 10 leeches per substrate); offspring per cocoon was analyzed with the cocoon as the unit of measurement (FLP, *n* = 17 cocoons; CTP, *n* = 18 cocoons). After adjustment for initial body weight of broodstock leeches, cocoon production did not differ significantly between substrates (Poisson GLM, *P* = 0.468), and offspring per cocoon showed no significant difference either (Mann–Whitney *U*-test, *P* = 0.424).

Nine of the 10 leeches on FLP and all 10 on CTP deposited at least one cocoon, whereas five and six, respectively, survived up to the end of the 75-day experiment; several animals therefore reproduced before dying later in the trial. Cocoon yield was nominally lower in leeches that died than in those that survived the full observation period (2.22 ± 0.46 vs. 3.45 ± 0.49 cocoons pooled across the two substrates), but the difference was not significant (Mann–Whitney *U*-test, *P* = 0.175).

The morphometric quality of cocoons did not differ between the two viable substrates on any measured trait (Figure 3). Cocoon weight (1.11 ± 0.08 g on FLP vs. 1.18 ± 0.11 g on CTP, Mann–Whitney *U*-test: *P* = 0.982), external length (24.85 ± 0.69 mm vs. 24.04 ± 0.87 mm, Student's *t*-test: *P* = 0.475), internal length (20.49 ± 0.58 vs. 19.90 ± 0.82 mm, Student's *t*-test: *P* = 0.563), external diameter (16.44 ± 0.34 vs. 16.24 ± 0.49 mm, Student's *t*-test: *P* = 0.749), and internal diameter (12.94 ± 0.35 vs. 12.81 ± 0.44 mm, Student's *t*-test: *P* = 0.819) were statistically indistinguishable. Effects of various reproductive substrates on the reproduction efficiency and cocoon characteristics were given in Table 3.

**Figure 3:**
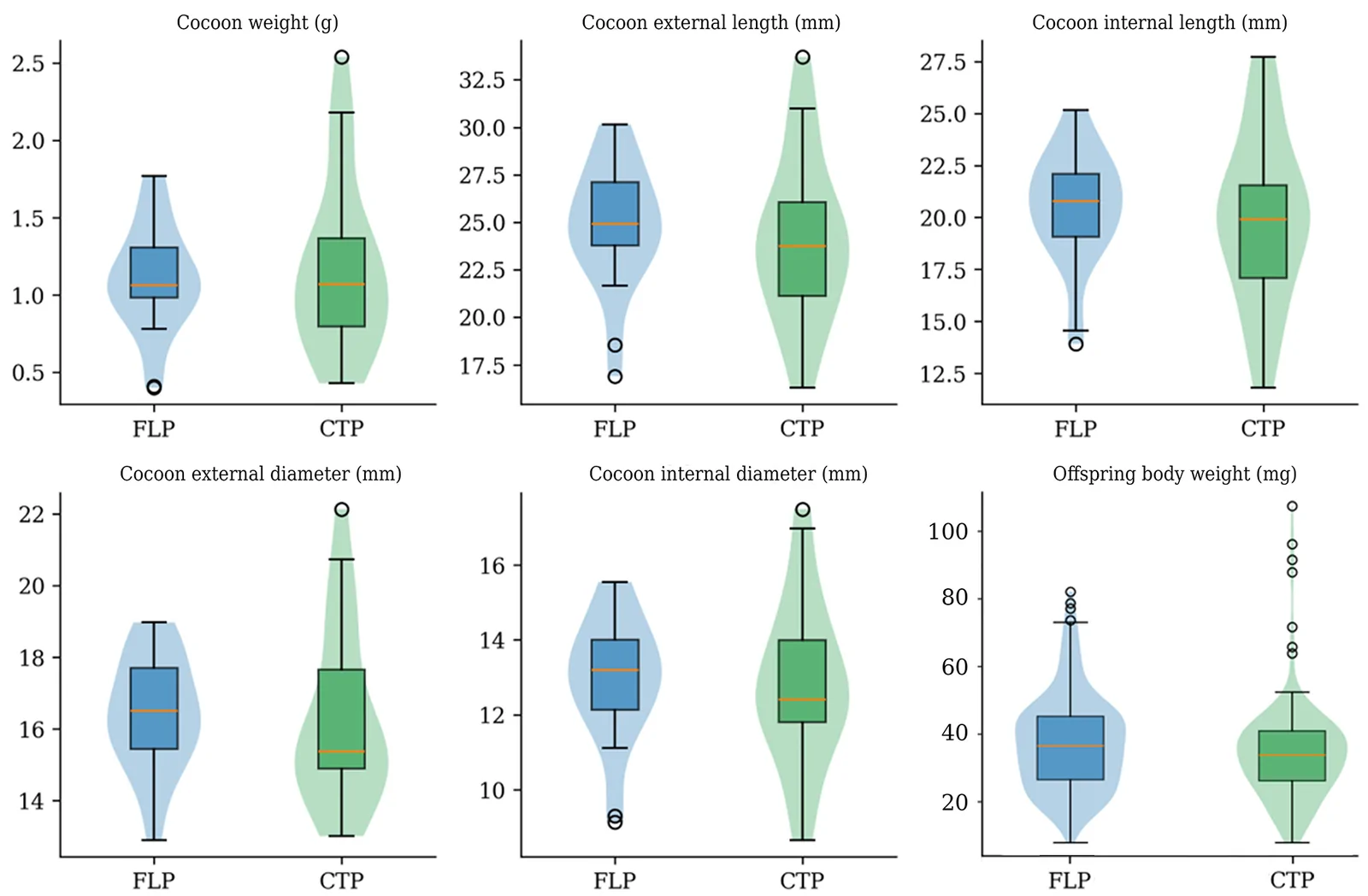
Comparison of cocoon characteristics and offspring body weight between viable peat substrates, fresh lake peat (FLP) and commercial terrestrial peat (CTP). Cocoon morphometrics were measured on the 46 cocoons recovered intact (FLP, *n* = 22; CTP, *n* = 24); offspring body weight was measured on all 366 individually weighed offspring (FLP, *n* = 218 offspring from 16 cocoons of 7 mothers; CTP, *n* = 148 offspring from 13 cocoons of 6 mothers). No significant differences were found between substrates for cocoon weight, external and internal length, and external and internal diameter (all *P* > 0.05). Similarly, offspring body weight was not significantly affected by the choice of substrate (Linear mixed model, *P* = 0.587). Analysis revealed that offspring derived from the FLP treatment exhibited a mean body mass (36.8 ± 1.0 mg) consistent with those from the CTP treatment (33.7 ± 1.2 mg).

**Table 3:** Effects of various reproductive substrates on the reproduction efficiency and cocoon characteristics of *Hirudo verbana*.

Substrate	Pregnancy duration (day)	Cocoon production per leech	Offspring production per cocoon	Offspring body weight (mg)	Cocoon morphometrics				
					Cocoon weight (g)	External length (mm)	Internal length (mm)	External diameter (mm)	Internal diameter (mm)
Commercial terrestrial peat (CTP)	46.9 ± 2.7	3.1 ± 0.4	12.2 ± 1.2	33.7 ± 1.2	1.18 ± 0.11	24.04 ± 0.87	19.90 ± 0.82	16.24 ± 0.49	12.81 ± 0.44
Fresh lake peat (FLP)	52.0 ± 2.9	2.7 ± 0.6	13.5 ± 1.6	36.8 ± 1.0	1.11 ± 0.08	24.85 ± 0.69	20.49 ± 0.58	16.44 ± 0.34	12.94 ± 0.35
Vermicompost (VC)	–	—	–	–	–	–	–	–	–
Commercial lake peat (CLP)	–	–	–	–	–	–	–	–	–
Sig.	0.384^a^	0.468^b^	0.424^a^	0.587^c^	0.982^a^	0.475^d^	0.563^d^	0.749^d^	0.819^d^

Values are expressed as mean ± standard error. Sample sizes differ by endpoint and reflect the biological unit measured: cocoon production per leech, *n* = 10 leeches per substrate; pregnancy duration, (FLP *n* = 7) and (CTP *n* = 8) leeches for which a complete deposition interval could be timed; cocoon morphometrics, (FLP *n* = 22) and (CTP *n* = 24) of the 46 cocoons recovered intact; offspring per cocoon, (FLP *n* = 17) and (CTP *n* = 18) cocoons; offspring body weight, *n* = 218 offspring from 16 cocoons of 7 mothers (FLP) and 148 offspring from 13 cocoons of 6 mothers (CTP). Hyphens (-) indicate parameters that could not be measured due to 100% broodstock mortality in commercial lake peat (CLP) and vermicompost (VC) prior to the reproductive phase. Superscript letters in the “Sig.” row indicate the statistical tests applied: ^a^Mann–Whitney U-test; ^b^Poisson GLM, ^c^Linear mixed model, ^d^Student's t-test.

### 3.4 Multivariate analysis of substrate physicochemical properties

Principal Component Analysis of the 14 physicochemical parameters produced a clear and interpretable ordination in which the first two components together explained 84.6% of the variance (PC_1_: 58.4%, PC_2_: 26.2%) (Figure 4). The lethal and viable substrates separated primarily along the first axis. VC occupied the extremely positive end of PC_1_ and was characterized by exceptionally high electrical conductivity, sodium, potassium, phosphorus, and the micronutrient metals copper and zinc, together with elevated pH. CLP lay at intermediate positive PC_1_ values and shared the elevated conductivity and metal signature, being especially high in manganese, copper, and zinc. The two viable substrates fell on the negative side of PC_1_: FLP was distinguished by high calcium, iron, and magnesium and by the lowest electrical conductivity of all substrates, while CTP separated along PC_2_ through its high organic matter, total nitrogen, and moisture content.

**Figure 4:**
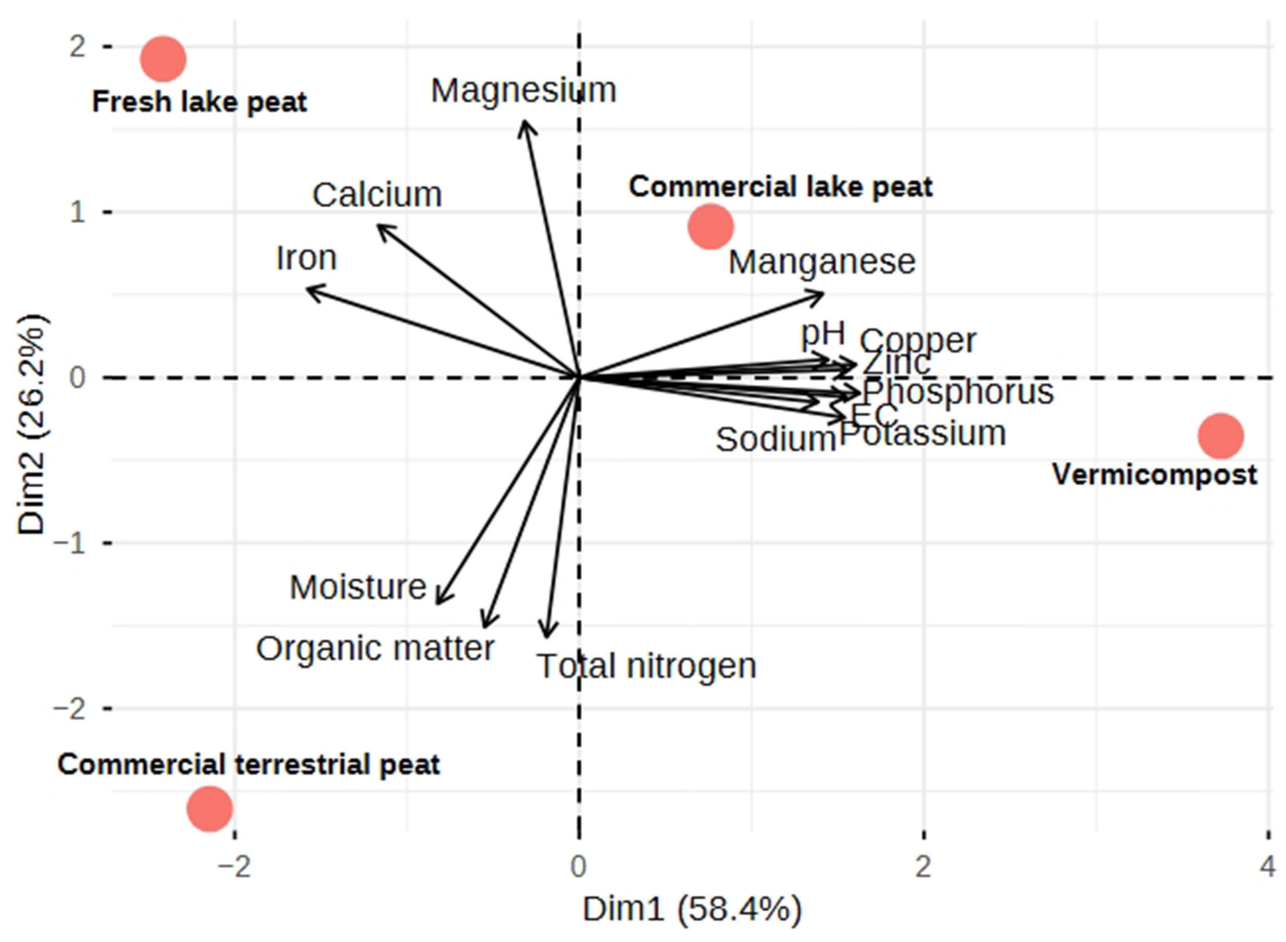
Principal Component Analysis of the physicochemical characteristics of the four reproductive substrates. PC_1_ and PC_2_ explained 58.4 and 26.2% of the total variance, respectively (84.6% cumulative). The analysis included the results of 14 different physicochemical analyses of four different substrates. The substrates were clearly separated according to their physicochemical profiles, with vermicompost (VC) and commercial lake peat (CLP) associated with higher electrical conductivity, pH, and mineral concentrations, whereas fresh lake peat (FLP) and commercial terrestrial peat (CTP) were associated with lower conductivity and distinct nutrient characteristics.

The clustered heatmap and associated hierarchical dendrogram of standardized physicochemical values (Figure 5) partitioned the experimental substrates into two primary clades, fundamentally separating the lethal commercial substrates (CLP and VC) from the viable peats (FLP and CTP). The parameter dendrogram further grouped the variables into distinct functional clusters that drove this broad separation. The lethal clade (VC and CLP) was distinctly characterized by intense positive z-scores for ionic and metal stressors. VC exhibited extreme relative elevations in phosphorus, sodium, potassium, and pH (*z* = 1.5). Furthermore, both VC and CLP shared strongly elevated profiles for manganese, electrical conductivity, copper, and zinc (*z* = 0.7–1.0).

**Figure 5:**
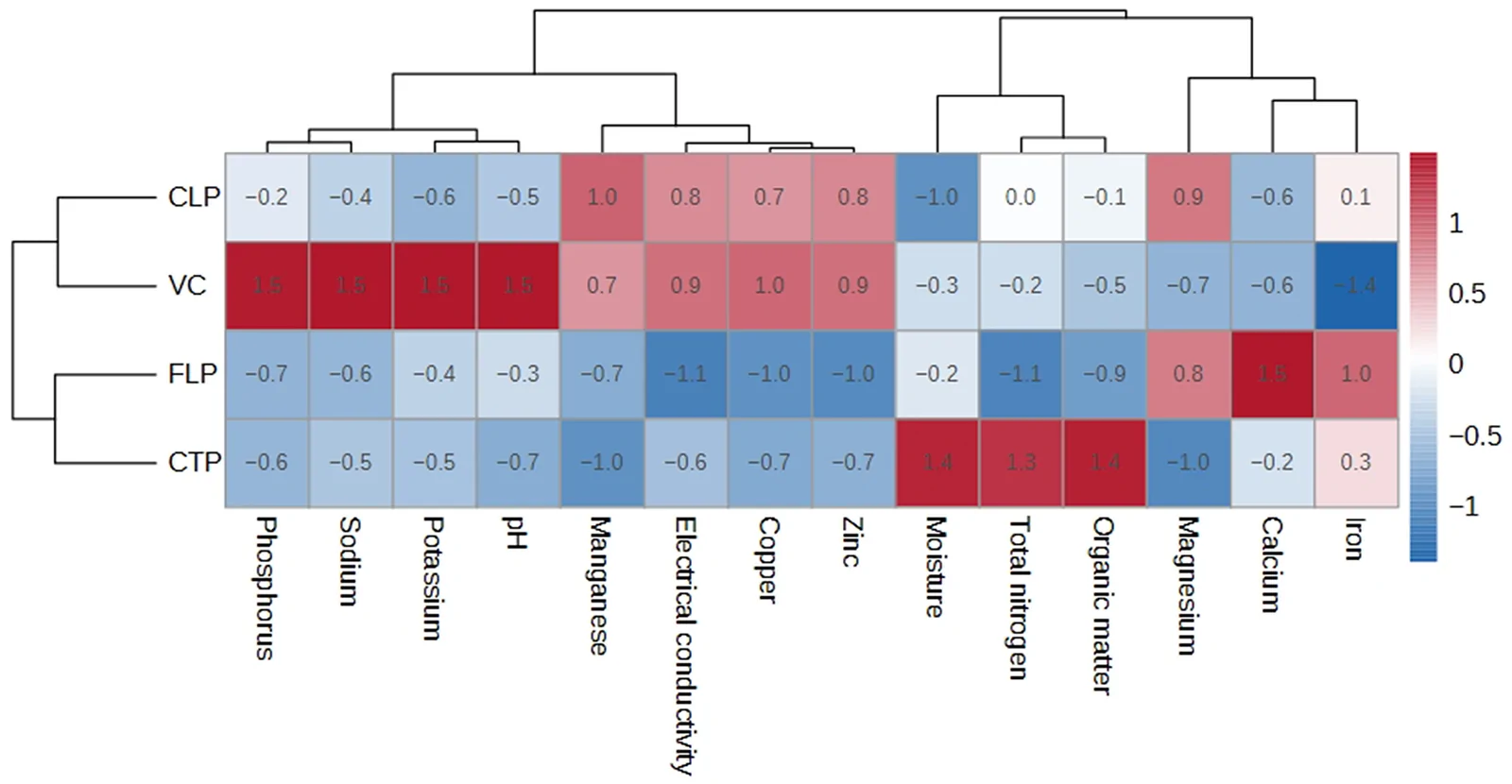
Clustered heatmap and hierarchical dendrogram of the standardized physicochemical profiles for the tested reproductive substrates. The dendrogram bifurcates the substrates into two primary clades: lethal (VC and CLP) and viable (FLP and CTP) substrates. Color gradients indicate standardized z-scores (blue: low; red: high). The analyses included the results of 14 different physicochemical analyses of four different substrates. The lethal clade is primarily driven by elevated ionic and heavy metal stressors (electrical conductivity, Mn, Cu, Zn), with VC also exhibiting extreme P, Na, K, and pH levels. Conversely, the viable clade features either high organic content, total nitrogen, and moisture (CTP), or elevated Ca and Fe (FLP). Ultimately, this clustering visually confirms that acute substrate-induced mortality is driven by osmotic and ionic stress, rather than organic content.

Within the viable clade, the substrates bifurcated reflecting their divergent compositional signatures. CTP was heavily characterized by high organic matter, total nitrogen, and moisture (*z* = 1.3–1.4). Conversely, FLP was defined by its minimal heavy metal and salinity loads (*z* ≤ −1.0), alongside elevated calcium (*z* = 1.5) and iron (*z* = 1.0). Ultimately, the tight clustering of heavy metals and salinity markers with the lethal substrates confirms that osmotic and ionic stress, rather than organic content, were the proximate drivers of substrate-induced mortality. Physicochemical characterization of the four reproductive substrates used in the study were given in Table 1.

## 4 Discussion

### 4.1 Substrate identity as a first-order determinant of reproductive success

The central finding of this study is unambiguous: the substrate provided to *H. verbana* during the reproductive period is a decisive determinant of survival, and two of the four commercially available substrates tested were acutely and completely lethal. VC and CLP killed every leech within one to 4 days and produced no cocoons at all, whereas FLP and CTP supported survival and normal reproduction. To our knowledge, this provides the first experimental evidence that a standard, commercially available leech aquaculture material can itself be lethal. The magnitude of the effect, a shift from complete survival of the reproductive cohort to complete mortality within days depending solely on the choice of reproductive substrate, is larger than the effects reported for most other husbandry variables in this species, including broodstock density (16), maternal age (18), and blood meal composition (13, 20), and it therefore deserves to be treated as a first-order control point in the design of leech aquaculture protocols.

### 4.2 Ionic and osmotic loading as the proximate mechanism

The underlying physiological mechanism driving this sharp contrast in survival rates has been clearly elucidated through the physicochemical profiling of the substrates. The lethal and viable substrates separated cleanly along the first principal component, which was defined by electrical conductivity, sodium, potassium, and the metals copper and zinc, and the ranking of substrates along this axis matched the ranking of mortality. This points to osmotic and ionic loading, rather than organic richness, as the proximate cause of death. The interpretation is physiologically coherent. Medicinal leeches are freshwater animals that regulate their internal osmotic and ionic environment against a dilute external reproductive substrate, and prolonged contact with a substrate whose pore water carries an electrical conductivity near 10 dS/m represents a severe osmotic challenge. Electrical conductivity is a well-established integrative proxy for soluble salt concentration in soils and organic media (24), and the FLP that best supported survival had by far the lowest conductivity of the four substrates. The osmotic interpretation is consistent with the wider ecology of freshwater, in which rising salinity and conductivity reduce invertebrate survival and restructure communities (35, 36), and with the substantial metabolic cost that ion regulation imposes on freshwater taxa held in strongly hypotonic media (37). Critically for a study of reproductive performance, this cost is not paid in survival alone. For instance, in certain freshwater invertebrates exposed to a rising salinity gradient, elevated osmoregulatory demand translated directly into reduced developmental and reproductive output (38).

The simultaneous presence of high conductivity and high levels of sodium, copper, and zinc in these substrates is not merely coincidental; rather, it reflects a well-known dynamic in which increased ionic strength mobilizes the metals and alters their bioavailability in aquatic systems (39). The elevated copper and zinc in the lethal substrates provide a second, complementary route to toxicity, because both metals are lethal to *H. verbana* at concentrations of the order encountered here, as shown in dedicated acute toxicity studies (25, 40). The appearance of blood regurgitation predominantly on the high-conductivity, metal-rich media is consistent with an acute physiological crisis rather than a slow nutritional failure, and it offers a readily observable early warning sign of substrate incompatibility (26).

That the two lethal substrates were precisely those most heavily processed for horticulture is not coincidental. VC and CLP are amended and marketed to maximize plant nutrition, and the very properties that make them effective plant media, namely high available nutrient and salt content, are the properties that make them hostile to freshwater osmoregulators (41, 42). This creates a systematic trap for leech breeders, because the substrates that are most attractively packaged and most readily available through the horticultural supply chain are the ones most likely to be dangerous. The finding aligns with the ecological observation that wild *H. verbana* deposit cocoons in damp, organically rich but low-salinity wetland margins and even in the nests of waterfowl (30), microhabitats whose chemistry resembles the FLP used here far more than it resembles a fertilized horticultural product, and which correspond to the low-density natural populations from which broodstock are still drawn. It also complements work showing that the physical, porous character of the substrate governs leech attachment and locomotion (29), by adding a chemical dimension to the definition of a suitable reproduction substrate.

### 4.3 Equivalence of viable substrates and the role of broodstock condition

Among the two substrates that supported reproduction, performance was essentially equivalent, and this equivalence is as valuable as the lethality of the other two. The number of cocoons per leech, cocoon morphology, and the number of offspring per cocoon did not differ between the FLP and CTP groups. Furthermore, individual larval weight was also comparable between the FLP (36.8 ± 1.0 mg) and CTP (33.7 ± 1.2 mg) groups, with no significant difference observed (*P* = 0.587). Given this complete equivalence across all measured reproductive parameters, our findings suggest that once a substrate clears the basic threshold of low salinity and low metal loading, reproductive output is buffered against further variation in the substrate, and producers are free to choose between a naturally collected and a commercially available low-salinity peat on grounds of cost, biosecurity, and convenience rather than reproductive performance. The CTP has the additional advantage that it delayed the onset of morphological deformation relative to FLP, and that it avoids the biosecurity risks and collection pressure associated with harvesting substrate from natural leech habitats. Because segmental and morphological deformations of this kind reflect measurable degradation of tissue integrity at the histological level (43, 44), a substrate that postpones their onset is likely to preserve broodstock condition and reuse value over a longer horizon. The reproductive figures obtained here also align closely with the only prior study to compare rearing media for this species, in which peat supported 3.13 cocoons per leech and 11.81 offspring per cocoon (32), values almost identical to the 3.1 cocoons per leech and 12.2 offspring per cocoon recorded on CTP in the present work. This concordance across independent laboratories strengthens confidence that low-salinity peat represents a stable and repeatable baseline for leech reproduction, and the output falls within the range documented for *H. verbana* in comparative studies of the genus (15). The association between initial body weight and cocoon number points in the same direction as the established understanding that reproductive output in *Hirudo* is governed chiefly by the size and condition of the broodstock (18), and it argues for conditioning leeches to a high body mass before the reproductive phase regardless of the substrate ultimately used.

### 4.4 Welfare and ethical considerations

The medicinal leech (*Hirudo verbana*), an invertebrate annelid, falls outside the scope of EU Directive 2010/63/EU and the relevant Turkish national legislation on the protection of animals used for scientific purposes; therefore, no specific institutional ethics approval was required for this study (Section 2.6). However, the absence of a legal requirement does not eliminate the practical welfare question raised by these results; this question concerns whether a routine and currently unevaluated rearing decision would cause preventable harm to animals kept in commercial and research settings.

The most obvious welfare-related finding here is also the least ambiguous. Two of the four routinely available commercial substrates killed the entire cohort within two to 4 days. No validated welfare index was required to detect this; it represents a complete and rapid rearing failure resulting from a substrate selection that producers currently make without any prior assessment method. Consequently, it appears that the damage can be prevented at a negligible cost, since the characteristic that distinguishes lethal substrates from viable ones can be measured with an inexpensive handheld device before broodstock leeches are introduced.

The two sublethal endpoints recorded here require more careful interpretation. Blood regurgitation was observed more frequently in lethal substrates (4/10 in VC and 3/10 in CLP; 1/10 in FLP and none in CTP) and this pattern paralleled the mortality rate, however, the difference was not statistically significant (*P* = 0.140), and the study, conducted with 10 animals per group, had limited power to detect a difference of this magnitude. Morphological deformation could be assessed only in two live substrates, as the animals in the VC and CLP groups died before deformities developed; there was no difference in prevalence between FLP and CTP (*P* = 0.650), but the mean onset was later in the CTP group (54.0 ± 7.7 days) compared to the FLP group (27.4 ± 6.8 days). Nevertheless, both endpoints are worth routinely recording: such deformities correspond to a measurable change at the tissue level (43, 44), and regurgitation can be directly observed during daily checks (45); therefore, together they offer a low-cost method for monitoring the condition of the broodstock. The exploratory association between a later onset of deformation and a higher cocoon yield supports this use, since it indicates that the timing of deformation tracks the reproductive capacity of the animal rather than merely its appearance.

Broodstock ended the trial having lost roughly 60%−70% of the body weight with which they started it. Two features of this loss argue against reading it as a substrate-attributable welfare failure. It did not differ between the two viable substrates (*P* = 0.082), so it neither discriminates between them nor can be attributed to substrate quality; and its magnitude is characteristic of a reproductive cycle in this species, broodstock *H. verbana* having been reported to lose 59.21 ± 8.90% of their body weight over a reproductive period (34) and 63 ± 8% or 40 ± 22% depending on the blood source used for conditioning (13). The values recorded here, 58.7 ± 5.3% on FLP and 67.3 ± 4.3% on CTP, fall squarely within that published range. FLP and CTP are therefore viable in the sense that broodstock survive the reproductive cycle and reproduce normally on them, and the operational implication concerns the post-reproductive refeeding period rather than the choice between these two peats.

It must be acknowledged that welfare assessment in leeches relies on such proxy indicators rather than validated criteria, and that drawing inferences about the subjective experience of an aquatic invertebrate goes far beyond what these data can support (46). What the current results support is narrower and more practical: an unregulated and routinely performed maintenance protocol can determine whether the broodstock will survive, and screening substrates before use eliminates this risk.

### 4.5 Practical recommendations for leech breeders

Taken together, these results yield a simple and actionable recommendation for leech breeders. Reproduction substrates should be screened for salinity before use, and electrical conductivity provides an inexpensive, rapid, and quantitative criterion that a producer can measure with a handheld meter. Because conductivity depends on how the extract is prepared, the criterion proposed here refers specifically to a 1:5 (w/v) substrate-to-water extract, the basis on which all values in this study were determined. On the evidence presented here, substrates with electrical conductivity in the low range, exemplified by FLP (1.19 dS/m) and CTP (3.69 dS/m), are suitable for the reproductive phase, whereas heavily fertilized horticultural products with conductivity approaching 10 dS/m and elevated sodium, copper, and zinc are likely to be lethal and should be avoided. Adopting conductivity screening as a standard quality control step adds a single measurement to the existing practice and requires no changes to the cultivation system itself. Body weight loss over the reproductive period does not discriminate between the two acceptable substrates (*P* = 0.082) and therefore does not enter the screening criterion; it is instead an argument for scheduling a defined refeeding and recovery interval before broodstock are committed to a further cycle.

### 4.6 Limitation of the study

These findings were obtained from a single reproductive cycle, and validating electrical conductivity as a general screening criterion will require larger groups, repeated reproductive cycles, and a broader range of commercial substrates. Each substrate was characterized from a single composite sample, so the physicochemical profiles carry no estimate of within-substrate variability and the ordination and clustering in Figures 4, 5 are descriptive rather than inferential.

## 5 Conclusion

The choice of reproduction substrate is a first-order determinant of success in the culture of the Mediterranean medicinal leech, *H. verbana*. Two widely available, horticulturally processed media, VC and CLP, were acutely lethal and eliminated the entire reproductive cohort within days, whereas FLP and CTP supported survival and equivalent reproductive performance. The lethal substrates were distinguished by high electrical conductivity together with elevated sodium, copper, and zinc, which points to osmotic and ionic stress as the proximate cause of mortality and implicates the fertilization and processing that make these substrates suitable for plants but hostile to a freshwater osmoregulator. For practitioners, the results translate into a single, inexpensive rule: measure electrical conductivity and reject high-salinity substrates before committing broodstock. Embedding this screening step into leech aquaculture protocols would prevent a major and previously uncharacterized source of loss and would strengthen the role of captive reproduction in conserving this clinically important and threatened species.

## Data Availability

All raw data supporting the findings of this investigation have been deposited in the public Figshare repository (doi: https://doi.org/10.6084/m9.figshare.33089861).
